# Efficacy and safety of Hou Gu Mi Xi for peptic ulcer diseases: Study protocol for a randomized controlled trial

**DOI:** 10.1097/MD.0000000000016561

**Published:** 2019-07-19

**Authors:** Xiaofan Chen, Dongmei Yan, Jianhe Fang, Wenjun Liu, Heyun Nie, Nonghua Lv, Nian Fang, Jinhua Gong, Jianwei Yu, Yiping Jiang, Zhijun Liu, Huihu Gan, Ying Fu, Deping Yang, Yan Xiong, Dunju Liu, Ming Chen, Yanping Wang, Yang Wang, Xin Sun, Xu Zhou, Weifeng Zhu

**Affiliations:** a Evidence-based Medicine Research Center, Jiangxi University of Traditional Chinese Medicine, Nanchang, Jiangxi; b School of Food Science and Engineering, Inner Mongolia Agricultural University, Hohhot, Inner Mongolia; c Department of Gastroenterology, the First Affiliated Hospital of Nanchang University; d Department of Gastroenterology, the Third Affiliated Hospital of Nanchang University; e Department of Gastroenterology, Jiujiang No.1 People's Hospital; f Department of Spleen, Stomach, Liver and Gallbladder Diseases, the Affiliated Hospital of Jiangxi University of Traditional Chinese Medicine; g Department of Gastroenterology, Xinyu People's Hospital; h Department of Spleen, Stomach, Xinyu Hospital of Traditional Chinese Medicine; i Department of Traditional Chinese Medicine, the Second Affiliated Hospital of Nanchang University; j Department of Gastroenterology, Nanchang Hospital of Integrated Traditional Chinese and Western Medicine; k Internal medicine 1, Nanchang Hongdu Hospital of Traditional Chinese Medicine; l Department of Gastroenterology, the Nanchang Third Hospital; m Department of Spleen, Stomach, Jiujiang Hospital of Traditional Chinese Medicine; n Chinese Evidence-based Medicine Center, West China Hospital, Sichuan University, Chengdu, Sichuan, China.

**Keywords:** dietary formula, Hou Gu Mi Xi, peptic ulcer disease, randomized controlled trial, traditional Chinese medicine

## Abstract

**Background:**

Peptic ulcer disease (PUD) is a major burden worldwide. Several challenges remain with standard Western treatment of PUD, such as persistent weakness, fatigue, and relapse. A dietary traditional Chinese medicine (TCM) formula, Hou Gu Mi Xi (HGMX), has been developed as a complementary treatment for PUD.

**Aims:**

This multicenter, double-blind, randomized controlled trial will assess efficacy and safety of HGMX in patients with PUD.

**Methods:**

Three hundred sixty eligible patients will be assigned to receive HGMX, placebo, HGMX + rabeprazole or placebo + rabeprazole for 4 weeks after 2 weeks of standard Western treatment. This first step, with a 2 × 2 factorial design, will focus on assessing the main and interaction effects of HGMX and rabeprazole on ulcer healing. Then, rabeprazole will be stopped, and HGMX will be continued for up to 1 year. The second step, with a placebo-controlled design, will compare the long-term effects of HGMX and placebo. Extended follow-up with no treatment will continue for up to 2 years. Independent and paired *t* tests, Pearson *χ*^2^ test and the rank-sum test will be used to compare between-group differences. The *P* value will be adjusted using the O’Brien & Fleming method for multiple comparisons.

**Expected outcomes:**

The primary outcomes are total efficacy rate of PUD treatment, quality of ulcer healing, and changes in spleen qi deficiency symptoms. The secondary outcomes include ulcer area, PUD recurrence, *Helicobacter pylori* eradication rate, gastric function, body weight, and body mass index. Adverse events (AEs), severe AEs, treatment-related AEs, and withdrawal owing to AEs will be recorded to assess treatment safety.

**Discussion:**

The trial results will provide high-quality evidence for HGMX, as a complementary therapy, for the long-term management of PUD and will be valuable for the development of related guidelines and regulations.

**Trial registration:**

The protocol of this trial was approved in all research hospitals and was registered in ClinicalTrials.gov at October 25, 2017(No. NCT03320538).

## Abstract 2

Supplemental Digital Content is available in the text

## 1 Introduction

Peptic ulcer disease (PUD) is a common digestive system disease that mostly involves the stomach and duodenum, corresponding to a gastric ulcer (GU) and a duodenal ulcer (DU). PUD may occur at any age, but more than half of patients are diagnosed at an age of 30 to 49 years.^[1]^ The global annual incidence of PUD has been estimated to be 0.10% to 0.19% based on physician diagnosis, with an annual prevalence ranging from 2.6% to 4.1%.^[2]^ PUD is a major problem in China, in which the prevalence was up to 17.2% in 2010.^[3]^ The pathogenesis of PUD is chronic damage to the gastroduodenal mucosa, which is mainly attributed to *Helicobacter pylori* (*H pylori*) infection and the use of nonsteroidal anti-inflammatory drugs (NSAIDs)^[4]^; 72.2% of *H pylori*-positive patients and 25% of patients with long-term NSAID use ultimately develop PUD.^[3,5]^

The typical symptoms of PUD are chronic, rhythmic, and periodic upper abdominal pain, as well as distension, belching, and inappetence.^[6]^ PUD can progress to severe complications, including bleeding, perforation, pyloric stenosis, and even cancerization, which may have life-threatening outcomes.^[7]^ Routine treatment of PUD involves a combination of *H pylori* eradication, acid suppression (eg, proton pump inhibitors [PPIs]), and gastric mucosa protection (eg, bismuth agents).^[4]^ This regimen shows definite efficacy, with an ulcer healing rate >90%.^[8]^ However, there are still some concerns with the routine treatment. Gastrointestinal symptoms generally improve significantly after treatment, but systemic symptoms (such as weakness and fatigue) can persist.^[9]^ Moreover, PUD relapse is not uncommon in patients who fail to eradicate *H pylori* and exhibit poor ulcer healing.^[10]^

In China, combinations with traditional Chinese medicine (TCM) have become an important complement to prevent and solve the above conditions.^[11]^ A number of animal and human studies have shown that TCM therapies, such as Chinese herbal medicine and acupuncture, have anti-ulcer effects and can reduce the recurrence of ulcers.^[12–17]^ In TCM theory, most cases of PUD can be classified as spleen qi deficiency (SQD) syndrome, in which the patient's spleen (an abstract organ responsible for transporting and transforming food and water that differs from the organ in Western medicine) and qi (a kind of abstract energy that sustains life) are weakened by disease to the point that are unable to function normally, resulting in a range of gastrointestinal and systemic symptoms.^[18]^ Therefore, the TCM treatment of SQD syndrome emphasizes invigorating spleen and replenishing qi to improve immunity and self-healing, and integrated TCM and routine treatments are expected to have greater efficacy against PUD.

Shen Ling Bai Zhu San is a classic TCM formula originally documented in the Song Dynasty (1102 AD). More than 3000 years of ancient experience has shown that Shen Ling Bai Zhu San has the functions of invigorating spleen and replenishing qi and can be used to treat PUD.^[19]^ Animal studies have shown that Shen Ling Bai Zhu San regulates absorption, mucosal ultrastructure, and the flora balance in the gastrointestinal tract to improve the ability to self-repair ulcers.^[20,21]^ However, there is a lack of high-quality randomized controlled trial (RCT) evidence on the efficacy and safety of this formula for PUD. Moreover, the powder preparation of Shen Ling Bai Zhu San is difficult to take and tastes bad, which is disadvantageous for the long-term management of PUD.

Hou Gu Mi Xi (HGMX), a dietary TCM formula modified from Shen Ling Bai Zhu San, was developed in 2016. HGMX replaces Atractylodes (*Baizhu*), the only component in Shen Ling Bai Zhu San forbidden to use as food according to the regulations in China, with orange peel (*Jupi*) and is made into cornmeal with japonica rice and oats to improve patient compliance. As a result, HGMX is composed of 10 dietary herbs: ginseng (*Renshen*), tuckahoe (*Fuling*), coixenolide (*Yiyiren*), Chinese yam (*Shanyao*), lotus seed (*Lianzi*), Amomum (*Sharen*), Platycodon (*Jiegen*), white hyacinth bean (*Baibiandou*), liquorice (*Gancao*), and orange peel. In this formula, ginseng and tuckahoe play roles in invigorating qi and nourishing spleen; coixenolide, Chinese yam, lotus seed, and Amomum help improve the effects of ginseng and tuckahoe; and Platycodon, white hyacinth bean, liquorice and orange peel assist in promoting lung qi and regulating the waterway to improve the absorption of the herbs. The combination of these dietary herbs is expected to be efficacious at improving immunity and ulcer healing. In addition, HGMX should have a good safety profile because of its dietary property, and no adverse events (AEs) related to HGMX have been reported in the 3 years of post-market safety monitoring.

Therefore, we hypothesize that HGMX, in combination with routine treatments, will improve ulcer healing, increase the *H pylori* eradication rate, and reduce the recurrence rate in patients with PUD, with a good safety profile and good patient compliance. To verify this hypothesis, we plan to conduct a RCT in 11 tertiary hospitals.

## 2 Methods and analysis

This study is a multicenter, randomized, double-blind trial. The protocol was registered at Clinicaltrials.gov (Number: NCT03320538) on October 25, 2017, and we report the protocol according to the Standard Protocol Items: Recommendations for Interventional Trials (SPIRIT) criteria.

### 2.1 Site and participant recruitment

The participants will be enrolled from 11 hospitals in 3 cities in Jiangxi Province: the First Affiliated Hospital of Nanchang University, the Second Affiliated Hospital of Nanchang University, the Third Affiliated Hospital of Nanchang University, Jiangxi Hospital of Traditional Chinese Medicine, Nanchang Hongdu Hospital of Traditional Chinese Medicine, Nanchang Hospital of Integrated Traditional Chinese and Western Medicine, The Nanchang Third Hospital, Xinyu People's Hospital, Xinyu Hospital of Traditional Chinese Medicine, Jiujiang No.1 People's Hospital, and Jiujiang Hospital of Traditional Chinese Medicine. Trial enrolment will be advertised through oral promotion by researchers, hospital posters, and web page advertising. The recruitment was started at July 10, 2017 and is expected to be completed in July 2020.

### 2.2 Patient screening

Patients will be deemed eligible after screening according to the inclusion and exclusion criteria.

#### 2.2.1 Inclusion criteria

1. Diagnosed with GU or DU by endoscopy and ulcer area from 0.3 to 1.0 cm^2^ with no indications of bleeding or perforation.
2. Diagnosed with SQD according to the criteria in the Consensus on Integrated Traditional Chinese and Western Medicine, as follows:
  - Main spleen deficiency (SD) symptoms: poor appetite; stomach or abdominal distention; loose stools or diarrhea.
  - Main qi deficiency (QD) symptoms: physical fatigue and weakness; mental fatigue and taciturnity.
  - Secondary symptoms: loss of taste or hypodipsia; stomach or abdominal pain; stomach tightness; nausea or vomiting; abnormal bowel sounds; lean or puffiness; sallow complexion; powerless defecation; facial or limb oedema.
  - Tongue symptoms: pale, swollen, or scalloped tongue with a thin, white coating.
  Patients will be diagnosed with SQD if they meet: 2 main SD symptoms + 2 main QD symptoms; 2 main SD symptoms + 1 main QD symptom + 1 tongue symptom; or 1 main SD symptom + 1 main QD symptom + 2 secondary symptoms + 1 tongue symptom.
3. Aged from 18 to 70 years.

#### 2.2.2 Exclusion criteria

1. Complex peptic ulcer (ie, simultaneous GU and DU).
2. History of ulcer complications (eg, bleeding, perforation, pyloric stenosis, and precancerous lesions).
3. Indications of ulcer complications, including bleeding and perforation (Forrest stage I, IIa or IIb; positive stool occult blood; or ulcer area >1 cm^2^).
4. History of use of NSAIDs, theophylline, oral antibiotics, potassium supplements, or any intervention for PUD within the last 3 months.
5. Pregnant, breastfeeding, or planning to become pregnant within 2 years.
6. Allergic to any trial interventions.
7. Impaired liver or renal function, indicated by total bilirubin >35 μmol/L, alanine transaminase >2× upper limit of normal (ULN), aspartate aminotransferase >2× ULN, or serum creatinine >ULN.
8. Other severe diseases (e.g., severe mental disorders and cancer).
9. Unwilling to provide personal information.

### 2.3 Randomization and blinding

Patients who pass the eligibility assessment will be randomly and proportionally divided into four groups: Group A, B, C, and D. We generated a block randomization sequence using Microsoft Excel that was saved as enciphered data. For allocation concealment, we will adopt centralized random assignment. Independent researchers who do not participate in trial procedures other than the randomization process will recode the random sequence into continuous numbers and label the boxes of trial samples. Samples will be allocated sequentially to participants. In this way, no one will know the group into which the next participant will be assigned.

**Figure 1:**
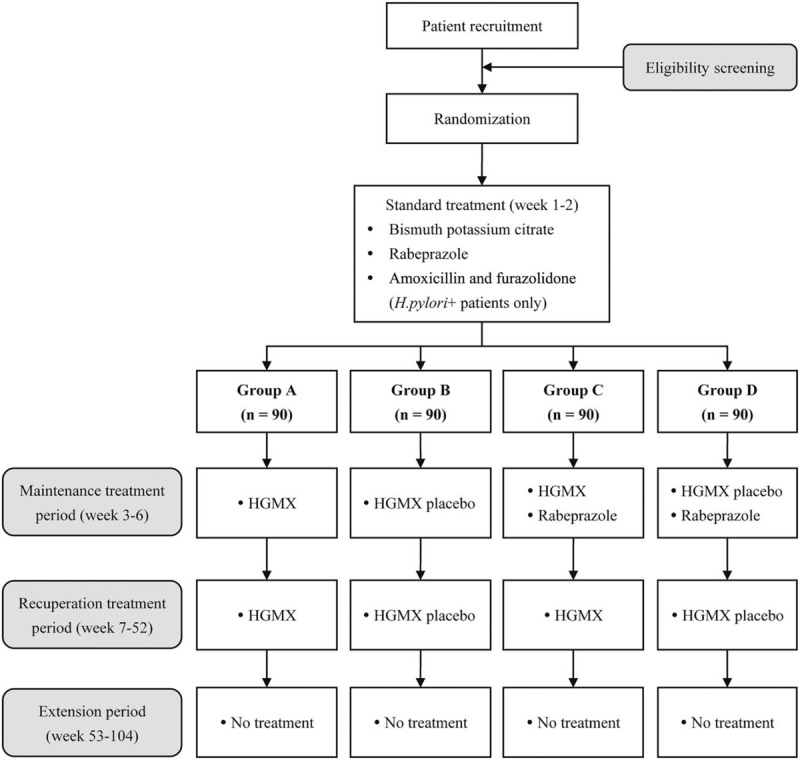
Study flow diagram. HGMX = Hou Gu Mi Xi.

This trial will have 3 intervention periods: standard treatment period, maintenance treatment period, and recuperation treatment period. The standard treatment period will be open-label. During the maintenance and recuperation treatment period, the clinicians, patients, outcome assessors, and data collectors will be blinded in terms of the assignment of HGMX or placebo but the administration of rabeprazole will be open-label. Except in instances of a severe AE, all the participants, medical providers, data collectors, and outcome reviewers will strictly adhere to the blinded de∗sign until the trial has been completed. In addition, an independent committee will supervise the allocation concealment and blinding, and if blinding is violated, the corresponding patient will be excluded. Figure 1 gives an overview of the study design and procedures.

### 2.4 Interventions and co-interventions

#### 2.4.1 Standard treatment period (weeks 1 to 2)

All eligible patients will receive standard Western treatment. Specifically, all patients will receive 20 mg rabeprazole b.i.d. and bismuth potassium citrate 0.3 g b.i.d. for 2 weeks. *H pylori*-positive patients will also receive 1 mg amoxicillin b.i.d. and 0.1 g furazolidone b.i.d. for 2 weeks to eradicate *H pylori*.

#### 2.4.2 Maintenance treatment period (weeks 3 to 6)

The patients in the 4 groups will receive the following intervention for 4 weeks as maintenance treatment to further facilitate ulcer healing.

1. Group A: HGMX 30 g b.i.d.
2. Group B: HGMX placebo 30 g b.i.d.
3. Group C: HGMX 30 g b.i.d. + rabeprazole 10 mg q.d.
4. Group D: HGMX placebo 30 g b.i.d. + rabeprazole 10 mg q.d.

HGMX is an oatmeal-like solid food (eaten dry or mixed with a liquid) that contains 10.1 g dietary herb material per 30 g. Table 1 presents the components of HGMX in detail. Compared with HGMX, the placebo looks the same (such as label, color and mass) but has minor differences in taste and smell that are hard to differentiate. HGMX and placebo are produced by Jiangzhong Pharmaceutical (group) Co., Ltd., and rabeprazole is produced by Shanghai Xinyi Pharmaceutical Corp. Ltd.

**Table 1:**
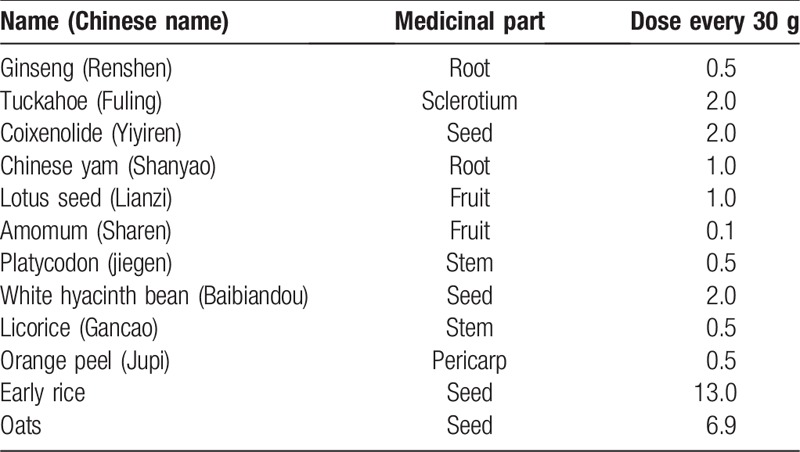
Specific components in Hou Gu Mi Xi.

#### 2.4.3 Recuperation treatment period (weeks 7 to 52)

All participants will enter the recuperation treatment period at the beginning of week 7. In this period, the doses of HGMX and placebo will remain unchanged (ie, 30 g b.i.d.) for all groups for a 46-week recuperation treatment. Rabeprazole will not be administered to the corresponding groups during this period.

#### 2.4.4 Extension period (weeks 53 to 104)

This is an observational period. All the interventions will be stopped, but follow-ups and outcome assessments will continue.

#### 2.4.5 Co-interventions

If participants have comorbidities not specified in the exclusion criteria, they will be allowed to receive targeted therapy, such as oral hypoglycemic drugs and insulin for diabetes, hypotensive drugs for hypertension, and antiallergy drugs for asthma.

Medicines that may damage the gastrointestinal tract, such as NSAIDs, theophylline and potassium supplements, and those that have a similar effect as our treatment, such as other PPIs, bismuth, and other Chinese herbal medicines, are prohibited.

### 2.5 Outcomes

#### 2.5.1 Primary outcomes

1. The total effective rate of PUD treatment will be calculated as (clinical cure + markedly effective + effective)/total number of participants. Efficacy will be assessed by Sakita–Miwa's method.^[22]^ First, ulcer healing status will be classified into 6 categories:
  - A1: the ulcer is round or elliptical, with a thick and white covering in the center; it can be accompanied by errhysis or blood clots and surrounded by obvious swelling, hyperemia, and oedema.
  - A2: the ulcer is covered by yellow or white patches, with no bleeding and less swelling and edema.
  - H1: the ulcer is healing; the coating of the ulcer becomes thin and subsides, hyperemia and edema disappear around the ulcer, and new capillaries appear.
  - H2: the ulcer becomes shallow and small; the surrounding mucosa plica is central to the ulcer.
  - S1: the white coating of the ulcer disappears, and the new ulcer mucosa is red.
  - S2: the new ulcer mucosa changes from red to white.
  Then, the efficacy will be ranked according to the following 4 levels based on ulcer rehabilitation and recovery:
  - Clinical cure: the ulcer completely heals with or without a scar.
  - Markedly effective: the ulcer heals to grade H2 or improves 2 grades.
  - Effective: the ulcer heals to grade H1 or improves by 1 grade.
  - Noneffective: no efficacy by endoscopic observation.
2. The quality of ulcer healing will be classified as follows by histopathological examination using Pan's criteria^[23]^:
  - Good: integral villus and epithelium, many glands, good morphology, many capillaries, and limited inflammatory cell infiltration.
  - Fair: short villus, incomplete or rough epithelium, fewer glands, structural distortion, few capillaries, and moderate inflammatory cell infiltration.
  - Poor: few new epithelial cells, poor epithelium integrity, poor morphology of villus and glands, very few capillaries, and a large number of inflammatory cells.
3. The changes in SQD symptoms will be assessed based on the Spleen Qi Deficiency Symptoms Grading and Quantifying Scale (SQD scale) score.^[19]^ This is a modified version of the classic scale that has a total of 15 items to assess 7 main symptoms and 8 secondary symptoms. Four aspects of SQD symptoms will be assessed, including severity, duration per episode, number of attacks per day, and number of affected days per week. The total score of each item is the sum of points for the 4 aspects in all items (items assessing the main symptoms receive double weight). The score ranges from 0 to 283 points, and a higher score indicates worse symptoms (see details in Supplementary file).

#### 2.5.2 Secondary outcomes

1. Ulcer area (cm^2^) measured by endoscopy.
2. Recurrence of PUD after standard and maintenance treatment, as measured by endoscopy.
3. Incidence of bleeding and perforation after standard and maintenance treatment, as measured by endoscopy.
4. *H pylori* eradication rate defined as a change in delta over baseline (DOB) value to <4 in the 13C-urea breath test during follow-up for *H pylori*-positive patients.
5. Gastric function evidenced by analysis of gastrin-17 (pg/mL), pepsinogen I (ng/mL), and pepsinogen II (ng/mL).
6. Changes in body weight (kilogram) and body mass index (kilogram per meter square).

#### 2.5.3 Safety outcomes

1. AEs, which will be assessed in 2 ways: significant abnormality (below or above twice the normal reference range) in the results of routine blood, urine or stool examinations, liver or kidney function tests, or electrocardiogram; AEs reported by clinicians and patients.
2. Severe AEs (SAEs), which are defined as AEs that are life-threatening or lead to admission to the intensive care unit, prolonged hospitalization, or physical or mental disability. Once an SAE occurs, the principal investigator will report it to the hospital institutional review board and submit the appropriate paperwork to the Food and Drug Administration, Department of Jiangxi Province, within 24 hours.
3. Treatment-related AEs, which must meet all the following criteria: the AE has reasonable time causality with treatment administration; comorbidities and co-interventions are not related to the AE; the symptoms of the AE improved or recovered when treatment was stopped; and the causality can be explained by pharmacological, biological, or phenomenological mechanisms.
4. Withdrawal owing to AEs.

### 2.6 Study visits

We will visit patients at baseline and at weeks 6, 12, 26, 52, and 104. In addition to the baseline measures of the outcomes, patients will undergo an eligibility assessment and provide demographic data, including gender, age, marital status, living situation, complications, medication history, and allergy history at baseline. During follow-up, we will examine ulcer histopathology at weeks 12 and 26; perform physical examinations, laboratory tests, and an electrocardiogram at weeks 12, 26, and 52; and examine *H pylori* infection and gastric function and perform gastroscopy at weeks 12, 26, 52, and 104. SQD symptoms, AEs, and concomitant treatments will be assessed or recorded at all follow-up visits. Table 2 gives an overview of the trial visits.

**Table 2:**
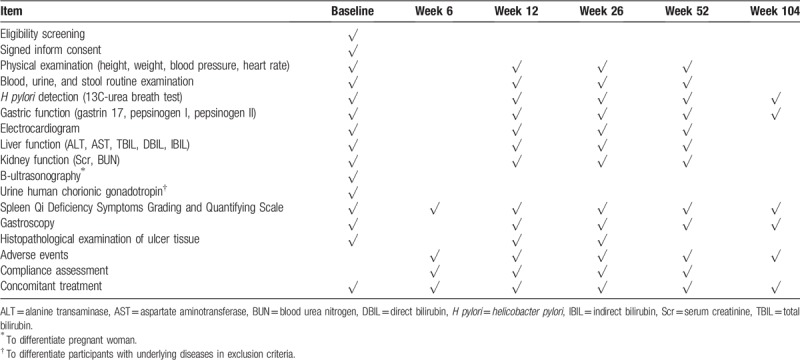
Schedule of trial visits.

### 2.7 Discontinuation and withdrawal

Anyone who reports any of the following conditions during follow-up will be classified as withdrawal, and the patient will discontinue the trial.

1. Acute recurrent ulcer (eg, acute bleeding and perforation).
2. SQD symptoms constantly worsen and require other interventions.
3. Patient took <80% of the prescribed dose or any prohibited drug.
4. Withdrawal owing to AEs.
5. Patient request for withdrawal.

### 2.8 Data management

Data collection and management will be carried out by a collaboration of research associates, coordinators and clinicians. All the investigators will undergo standard operating procedure (SOP) training based on the study protocol. During the trial, subject recruitment, intervention, follow-up, data collection and data management will be performed in strict accordance with the SOPs.

The clinicians will be responsible for the baseline and outcome examinations, including SQD symptom assessments, biological sample collections, and laboratory examinations. All these data will be entered on pilot-tested case report forms. The research coordinators will be student volunteers from Jiangxi University of Traditional Chinese Medicine and will be responsible for contacting patients and helping with communication between patients and doctors. We will assign to each study center a research associate selected from researchers at Jiangxi University of Traditional Chinese Medicine who will be responsible for monitoring the follow-up process, standard performance, patient compliance, and completeness of data collection. We will develop a specialized computer system for data entry that will support data query and verification. Once verified, data will be locked and accessible to only data analysts during the data analysis period. In special cases and emergency conditions, such as SAEs, the database will be temporarily accessible, and data for the corresponding participant will be unblinded.

### 2.9 Sample size estimation

The main objective of this trial is to assess the effects of HGMX compared with placebo on the total effective rate. Therefore, the sample size estimation is based on the formula for a superiority design.^[24]^ Because there are no previous studies available for reference, we estimated a *P* of .85 (total effective rate in the two groups receiving HGMX) and a *P*_0_ of .65 (total effective rate in the 2 groups receiving placebo) according to the results of our pilot survey and expert opinions. In addition, we adopted a type I error (α) of 0.05, a type II error (β) of 0.2, and a maximum drop-out rate of 15%. Based on these assumptions and our funding constraints, we computed a sample size of 360, with 90 in each group.

### 2.10 Data analysis

The primary outcome data will be analyzed using the intention-to-treat population, in which all patients will be maintained in their initial group assignment regardless of compliance and contamination, and missing data will be imputed using the last observation carried forward method. Meanwhile, we will also conduct a sensitivity analysis with the per-protocol population. The safety outcomes will be analyzed using a safety population including all participants who received treatment at least once. Mid-term and final data analyses will be performed at the end of week 52 and 104, respectively.

Normality will be detected using the Kolmogorov-Smirnov method. Normal continuous variables will be described using the mean and standard deviation and compared using a *t* test or analysis of variance. Non-normal continuous variables will be described using the median and interquartile range and analyzed with the Mann–Whitney *U* test. Categorical variables will be presented as the frequency and percentage and compared using Pearson *χ*^2^ test. The boundary of significance for the *P* value will be .05, which will be adjusted using the O’Brien & Fleming method for multiple comparisons.

According to our factorial design, we will explore whether there exists an interaction between HGMX and rabeprazole. If there are no significant interaction effects, we will compare the main effects of HGMX and rabeprazole. In addition, we will perform subgroup analysis stratified by type of PUD (GU vs. DU) and *H pylori* infection status (yes vs no). All statistical analyses will be conducted in Stata v14.0 software.

### 2.11 Trial monitoring

An independent data monitoring committee (DMC) will be found, which will be responsible to supervise the overall efficacy and safety of the trial. The DMC will have right to decide an interim data analysis and to stop the trial if obvious benefits or harms are observed.

### 2.12 Ethics approval

This trial protocol was approved by the Ethics Committee in the 11 research hospitals: Affiliated Hospital of Jiangxi University of Traditional Chinese Medicine (JZFYLL2017071406), First Affiliated Hospital of Nanchang University (2017YYLS028), Second Affiliated Hospital of Nanchang University (2017YYLS1011), Nanchang First Hospital (KYC2017302), Nanchang Third Hospital (2017KYLS001), Nanchang Hongdu Hospital of Traditional Chinese Medicine (KY-2017-004), Nanchang Hospital of Integrated Traditional Chinese and Western Medicine (2017YYLS001), Xinyu People's Hospital (2017LLS002), Xinyu Hospital of Traditional Chinese Medicine (2017YYLS1), Jiujiang No.1 People's Hospital (JJSDYRMYY-LL-2017-007), and Jiujiang Hospital of Traditional Chinese Medicine (JJSZYYY20170721).

## 3 Discussion

The main purpose of this study is to clarify the efficacy and safety profile of HGMX, a dietary TCM formula, in patients with PUD and SQD syndrome and assess their treatment compliance. The assessment at week 12 will focus on the main and interaction effects of HGMX and rabeprazole on the quality of ulcer healing, and the following assessments are designed to assess the long-term effects of HGMX compared with placebo on the total effective rate, quality of ulcer healing, SQD symptoms, and PUD recurrence rate. Owing to manufacturing limitations, we cannot produce a placebo for rabeprazole and thus cannot utilize a blinded design for rabeprazole treatment, which will be a main limitation of our study and may lead to potential bias due to placebo effects. Another underlying limitation is that we chose a self-modified scale that lacks confirmation of reliability and validity and thus may introduce measurement bias.

Dietary TCM formulas are widely used for chronic disease management in China. However, the use of dietary TCM formulas is challenged by the lack of high-quality research evidence and related regulations. To produce high-quality evidence to increase the clinical use of HGMX, we will conduct a series of RCTs involving participants with PUD, nonorganic gastrointestinal disorders (NOGDs), and radical gastrectomy for gastric cancer. These are the first RCTs committed to evaluating a dietary TCM formula, and they will hopefully establish an evidence-based clinical research model for dietary TCM formulas and be valuable for the development of related guidelines and regulations.

## Acknowledgments

The authors thank 10 student volunteers from Jiangxi University of Traditional Chinese Medicine (Honghong Ma, Pengen Qin, Dongzhiwei Zhang, Shicheng Ye, Wenqiang Qian, Haidong Zhou, Jinsheng Zeng, Qianqian Luo, Shunrong Chen, Mengxia Gao) for their great efforts on the patient enrollment.

## Author contributions

**Conceptualization:** Xu Zhou, Weifeng Zhu.

**Investigation:** Nonghua Lv, Nian Fang, Jinhua Gong, Jianwei Yu, Yiping Jiang, Zhijun Liu, Huihu Gan, Ying Fu, Deping Yang, Yan Xiong, Dunju Liu, Ming Chen.

**Supervision:** Jianhe Fang, Wenjun Liu, Heyun Nie, Yanping Wang.

**Writing – original draft:** Xiaofan Chen, Dongmei Yan, Yang Wang.

**Writing – review & editing:** Xin Sun, Xu Zhou, Weifeng Zhu.

## Supplementary Material

Supplemental Digital Content
