# Supplementary material for: Efficacy and safety of Hou Gu Mi Xi for peptic ulcer diseases: Study protocol for a randomized controlled trial
Source: Medicine (Baltimore). 2019 Jul 19;98(29):e16561. doi: 10.1097/MD.0000000000016561 (PMC6709288; doi:10.1097/MD.0000000000016561)
Supplement: Supplemental Digital Content [file medi-98-e16561-s001.docx]

**Spleen Qi Deficiency Symptoms Grading and Quantifying Scale**

| **Item** | **Option** |
| --- | --- |
| **1 Stomach pain^*^** | □_0_ None |
| Severity | □_1_ Mild □_2_ Moderate □_3_ Severe |
| Duration per episode | □_1_ < 0.5 hour □_2_ 0.5-1 hour □_3_ > 1 hour |
| Frequency per day | □_1_ Occasionally　□_2_ Sometimes　□_3_ Most of the time　□_4_ Persistently |
| Frequency per week | □_1_ < 1 day □_2_ 1 day □_3_ 2-3 days □_4_ 4-5 days □_5_ 6-7 days |
| **2 Stomach distension^*^** | □_0_ None |
| Severity | □_1_ Mild □_2_ Moderate □_3_ Severe |
| Duration per episode | □_1_ < 0.5 hour □_2_ 0.5-1 hour □_3_ > 1 hour |
| Frequency per day | □_1_ Occasionally　□_2_ Sometimes　□_3_ Most of the time　□_4_ Persistently |
| Frequency per week | □_1_ < 1 day □_2_ 1 day □_3_ 2-3 days □_4_ 4-5 days □_5_ 6-7 days |
| **3 Acid reflux^*^** | □_0_ None |
| Severity | □_1_ Mild □_2_ Moderate □_3_ Severe |
| Frequency per day | □_1_ Occasionally　□_2_ Sometimes　□_3_ Most of the time　□_4_ Persistently |
| Frequency per week | □_1_ < 1 day □_2_ 1 day □_3_ 2-3 days □_4_ 4-5 days □_5_ 6-7 days |
| **4 Abdominal distension^*^** | □_0_ None |
| Severity | □_1_ Mild □_2_ Moderate □_3_ Severe |
| Duration per episode | □_1_ < 0.5 hour □_2_ 0.5-1 hour □_3_ > 1 hour |
| Frequency per day | □_1_ Occasionally　□_2_ Sometimes　□_3_ Most of the time　□_4_ Persistently |
| Frequency per week | □_1_ < 1 day □_2_ 1 day □_3_ 2-3 days □_4_ 4-5 days □_5_ 6-7 days |
| **5 Abnormal stools^*^** | □_0_ None |
| Severity | □_1_ Unshapen stool □_2_ Loose stool □_3_ Diarrhea |
| Frequency per day | □_1_ 1 time □_2_ 2 times □_3_ 3 times □_4_ ≥ 4 times |
| Frequency per week | □_1_ < 1 day □_2_ 1 day □_3_ 2-3 days □_4_ 4-5 days □_5_ 6-7 days |
| **6 Fatigue and weakness^*^** | □_0_ None |
| Severity | □_1_ Mild □_2_ Moderate □_3_ Severe |
| Frequency per day | □_1_ Occasionally　□_2_ Sometimes　□_3_ Most of the time　□_4_ Persistently |
| Frequency per week | □_1_ < 1 day □_2_ 1 day □_3_ 2-3 days □_4_ 4-5 days □_5_ 6-7 days |
| **7 Loss of appetite^*^** | □_0_ None |
| Severity | □_1_ Mild □_2_ Moderate □_3_ Severe |
| Frequency per day | □_1_ 1 meal □_2_ 2 meals □_3_ 3 meals |
| Frequency per week | □_1_ < 1 day □_2_ 1 day □_3_ 2-3 days □_4_ 4-5 days □_5_ 6-7 days |

(*Continued*)

| **Item** | **Option** |
| --- | --- |
| **8 Stomach tightness** | □_0_ None |
| Severity | □_1_ Mild □_2_ Moderate □_3_ Severe |
| Duration per episode | □_1_ < 0.5 hour □_2_ 0.5-1 hour □_3_ > 1 hour |
| Frequency per day | □_1_ Occasionally　□_2_ Sometimes　□_3_ Most of the time　□_4_ Persistently |
| Frequency per week | □_1_ < 1 day □_2_ 1 day □_3_ 2-3 days □_4_ 4-5 days □_5_ 6-7 days |
| **9 Heart burning** | □_0_ None |
| Severity | □_1_ Mild □_2_ Moderate □_3_ Severe |
| Frequency per day | □_1_ Occasionally　□_2_ Sometimes　□_3_ Most of the time　□_4_ Persistently |
| Frequency per week | □_1_ < 1 day □_2_ 1 day □_3_ 2-3 days □_4_ 4-5 days □_5_ 6-7 days |
| **10 Belching** | □_0_ None |
| Severity | □_1_ Mild □_2_ Moderate □_3_ Severe |
| Frequency per day | □_1_ Occasionally　□_2_ Sometimes　□_3_ Most of the time　□_4_ Persistently |
| Frequency per week | □_1_ < 1 day □_2_ 1 day □_3_ 2-3 days □_4_ 4-5 days □_5_ 6-7 days |
| **11 Nausea and vomiting** | □_0_ None |
| Severity | □_1_ Mild □_2_ Moderate □_3_ Severe |
| Frequency per day | □_1_ Occasionally　□_2_ Sometimes　□_3_ Most of the time　□_4_ Persistently |
| Frequency per week | □_1_ < 1 day □_2_ 1 day □_3_ 2-3 days □_4_ 4-5 days □_5_ 6-7 days |
| **12 Powerless defecation** | □_0_ None |
| Severity | □_1_ Mild □_2_ Moderate □_3_ Severe |
| Frequency per day | □_1_ Occasionally　□_2_ Sometimes　□_3_ Most of the time　□_4_ Persistently |
| Frequency per week | □_1_ < 1 day □_2_ 1 day □_3_ 2-3 days □_4_ 4-5 days □_5_ 6-7 days |
| **13 Mental fatigue and taciturnity** | □_0_ None |
| Severity | □_1_ Mild □_2_ Moderate □_3_ Severe |
| Frequency per day | □_1_ Occasionally　□_2_ Sometimes　□_3_ Most of the time　□_4_ Persistently |
| Frequency per week | □_1_ < 1 day □_2_ 1 day □_3_ 2-3 days □_4_ 4-5 days □_5_ 6-7 days |
| **14 Sallow complexion** | □_0_ None |
| Severity | □_1_ Mild □_2_ Moderate □_3_ Severe |
| Frequency per day | □_1_ Occasionally　□_2_ Sometimes　□_3_ Most of the time　□_4_ Persistently |
| Frequency per week | □_1_ < 1 day □_2_ 1 day □_3_ 2-3 days □_4_ 4-5 days □_5_ 6-7 days |

(*Continuous*)

| **Item** | **Option** |
| --- | --- |
| **15 Loss of taste and hypodipsia** | □_0_ None |
| Severity | □_1_ Mild □_2_ Moderate □_3_ Severe |
| Frequency per day | □_1_ Occasionally　□_2_ Sometimes　□_3_ Most of the time　□_4_ Persistently |
| Frequency per week | □_1_ < 1 day □_2_ 1 day □_3_ 2-3 days □_4_ 4-5 days □_5_ 6-7 days |

*: primary symptoms

Reference for assessing severity of symptoms or signs:

Mild: The symptoms or signs are not obvious and patients cannot felt without reminding;

Moderate: The symptoms or signs are obvious but do not affect patients’ living and work;

Severity: The symptoms or signs are very obvious and affect patients’ living and work.
